# Impact of Chorea on Self-care Activity, Employment, and Health-care Resource Use in Patients with Huntington’s Disease

**DOI:** 10.36469/001c.24620

**Published:** 2021-06-21

**Authors:** Daniel O. Claassen, Jonathan DeCourcy, Jennifer Mellor, Charlotte Johnston, Ravi G. Iyer

**Affiliations:** 1 Vanderbilt University Medical Center, Nashville, TN, USA; 2 Adelphi Real World, Manchester, UK; 3 Teva Pharmaceuticals, West Chester, PA, USA

**Keywords:** health services needs and demand, employment, quality of life, activities of daily living, chorea, huntington’s disease

## Abstract

**Background:** Chorea is recognized as a prototypic motor feature of Huntington’s disease (HD), but its effect on health-related quality of life (HRQoL) has not been fully explored. This study describes the impact of chorea on HRQoL in patients with HD.

**Objective:** To determine the impact of HD-related chorea on employment, self-care activities, activities of daily living, and health-care resource utilization (HCRU).

**Methods:** Data were drawn from the Adelphi HD Disease Specific Programme, a real-world point-in-time survey of 144 neurologists and 427 patients in the United States between July and October 2017. HD patients with and without chorea were identified and examined for differences in employment status, reasons for employment changes, self-care activities, and modifications to cope with involuntary movements. Bivariate tests and inverse probability weighted regression adjustment methods were used to determine differences in outcomes between patients with and without chorea.

**Results:** HD patients with (n=287) and without (n=140) chorea were identified. Patients with chorea were less likely to be employed full-time (16.7% vs 25.7%; *P*<0.04) and more likely to be on long-term sick leave (17.4% vs 5.0%; *P*<0.01). The onset of motor symptoms in HD-related chorea patients coincided with a change in employment status (42.7% vs 20.8%; *P*<0.01). Among those still working (n=145), more than two-fifths of patients with chorea required changes to their workplace and required these changes more frequently (45% vs 17%; *P*<0.001). HD patients with chorea required aid to help them get around significantly more frequently than those without chorea (55% vs 34%; *P*<0.001).

**Discussion:** These results demonstrate that HD patients with chorea experienced greater negative impact to employment, self-care activities, and HCRU than patients without chorea experienced. These patients were more likely to stop working due to motor, cognitive, and behavioral symptoms; require modifications in the home and workplace; and need more assistance from caregivers than patients without chorea.

**Conclusions:** Patients with HD-related chorea have greater detriments to emotional, interpersonal, and professional functioning that could be improved by reducing chorea.

## INTRODUCTION

Symptoms of Huntington’s disease (HD) typically manifest in the third to fifth decades of life through the development of motor, cognitive, and psychiatric manifestations.^1–3^ Chorea is recognized as one of the prototypic motor features of HD, characterized by involuntary hyperkinetic movements.^4^ Chorea is estimated to be present in about 90% of HD patients during the course of a lifespan.^5^ Initially, chorea symptoms may be subtle and not associated with functional impairments, presenting as muscle twitches or fidgetiness.^6,7^ As the disease progresses, chorea can worsen, with involuntary movements increasing in both amplitude and frequency leading to functional impairments.^6,7^ The hyperkinetic symptoms related to chorea can affect any muscle, leading to falls, poor dexterity, and difficulty swallowing.^8–10^ Chorea also disrupts the ability of patients with HD to complete daily tasks independently, leading to the requirement for full-time care.^9,11,12^ Furthermore, the movements caused by chorea are often stigmatizing (as they are commonly mistaken for intoxication)^13^ and can be associated with lower overall health-related quality of life (HRQoL),^14–17^ emphasizing the need to treat this detrimental symptom.

Life expectancy in HD patients varies, but is up to 20 years after motor diagnosis.^18^ Consequently, interventions that can keep patients employed, socially engaged, and independent are desirable. Chorea is a motor symptom that is amenable to treatment, but its effect on HRQoL has not been fully explored. There is a lack of real-world evidence describing the effect of chorea on patients with HD in relation to their employment, self-care activities, and independence. We sought to assess the impact of chorea on HD patients’ functionality in everyday tasks, as well as on their employment and health-care resource utilization (HCRU). This study describes real-world data capturing the impact of chorea on HD patients in the United States, and the burden and impact of this disease on everyday life.

## METHODS

### Survey Design

Data were drawn from the Adelphi HD Specific Programme (DSP™) conducted between July and October 2017 in the United States. A total of 427 patients with HD were included in the study along with their 144 consulting neurologists. The DSP™ was a large, point-in-time survey of physicians and their patients presenting in a real-world clinical setting, describing current disease management, disease-burden impact, and associated treatment effects (clinical and physician-perceived). A complete description of the methods of the survey has been previously published and validated.^19–21^ Physicians were instructed to complete a record form for their patients with HD, either retrospectively from their recent records or prospectively from their next consult. This physician-reported form contained detailed questions on patient demographics, consultation and diagnosis history, clinical assessments, treatment history, HCRU, and concomitant conditions, using the Charlson Comorbidity Index (CCI),^22^ which accounts for the number and severity of comorbid conditions. The physician-reported survey forms included set questions with prespecified options that were single or multiple choice as appropriate. Completion of the physician-reported survey was undertaken through consultation of existing patient clinical records, as well as the judgment and diagnostic skills of the respondent physician, which is entirely consistent with decisions made in routine clinical practice.

### Participating Physicians and Patients

Physicians were eligible to participate in this study if they were personally responsible for treatment decisions and management of patients with HD. Participating physicians completed records on patients who were >18 years old, had a physician-confirmed diagnosis of HD, and had consulted the physician immediately before beginning the study or during the duration of the study. It should be noted that the study was designed to facilitate understanding of real-world clinical practice, and thus physicians could only report on data they had at the time of consultation. No additional tests, treatments, or investigations were performed as part of this study. Missing data were not imputed; therefore, the base of patients for analysis could vary from variable to variable and is reported separately for each analysis.

### Statistical Analysis

Basic descriptive statistics, such as means and proportions not requiring statistical comparisons, were carried out and derived using the software IBM® SPSS® Data Collection Survey Reporter 7. Any analyses that required statistical comparisons were conducted using STATA® Version 10 (StataCorp LP, College Station, Texas) and SPSS® Version 15 (SPSS Inc., Chicago, Illinois). At the bivariate level, standard parametric and nonparametric statistical tests were performed that were appropriate for the data type and comparison group. Categorical variables were compared using Fisher’s exact test, and numeric variables were compared using the student’s *t* test. Each test resulted in a *P*-value, which indicated a statistically significant difference between groups if the value was <0.05.

To account for confounding factors that may influence the bivariate analysis, inverse probability weighted regression adjustment (IPWRA) analysis was also performed. IPWRA is a doubly robust method, which uses a logistic regression model to determine a propensity score of how likely a patient is to experience chorea or not, given a prespecified list of covariates (in this case, age, body mass index [BMI], sex, and CCI). The inverse of the propensity score is used to weight patients in the two groups: with chorea versus without chorea. The resulting treatment-specific weighted means or proportions of clinical characteristics are reported, accounting for differences in likelihood of having chorea, according to the covariates, with patients predicted as unlikely to have chorea given a higher weight, and those likely to have chorea given a lower weight. *P*-values for average treatment effect (ATE) are also reported.

Due to the nature of IPWRA analysis, bases between analyses vary, as any “Don’t know” responses to outcomes are excluded.

### Ethical Compliance Statement

A complete description of the methods of the study has been previously published and validated.^19–21^ Using a check box, patients provided informed consent for use of their anonymized and aggregated data for research and publication in scientific journals. Data were collected such that patients and physicians could not be identified directly; all data were aggregated and de-identified before receipt. This research also obtained ethics approval from the Freiburg Ethics Commission International (Study code AG8260). Data collection was undertaken in line with European Pharmaceutical Marketing Research Association^23^ guidelines, and as such it does not require ethics committee approval. The study was performed in full accordance with relevant legislation at the time of data collection, including the US Health Insurance Portability and Accountability Act of 1996 and the Health Information Technology for Economic and Clinical Health Act.^24,25^

## RESULTS

### Descriptive Patient Demographics

The survey included 144 neurologists who completed forms on a total of 427 HD patients in the United States. These patients were then categorized into two groups: those with chorea (n=287) and those without chorea (n=140; **Figure S1**). Patients with chorea were defined as those suffering from any of the following symptoms: involuntary movement of limbs, clumsiness, fidgeting, or twitching.

These groups were used to compare the burden and impact that chorea has on employment and a patient’s everyday activities. There was a similar split between male and female patients with versus without chorea (male: 63% vs 64%, respectively; female: 37% vs 36%, respectively). A significant difference was found between the ages of the patients in the two groups (*P*=0.013; **Table S1**); therefore, IPWRA was used to account for age, BMI, sex, and impact of comorbid conditions using the CCI for the following analyses.

### Impact of Chorea on Employment (IPWRA Analysis)

**Figure 1.**
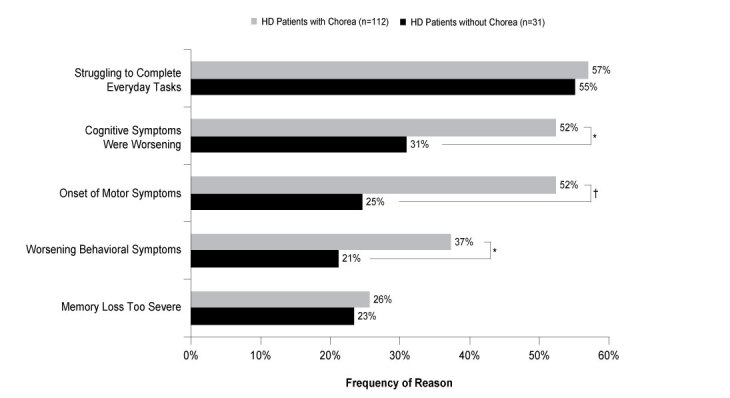
Reasons HD Patients with and without Chorea Stopped Working Abbreviations: HD, Huntington’s disease. *P<0.05; †*P*<0.01.

An analysis was performed on patients who were on long-term sick leave, retired, or unemployed, with known reasons for stopping employment. This included 112 patients with chorea and 31 patients without chorea. Of the patients with chorea, more than four-fifths were out of work due to HD-related symptoms versus just over two-thirds of patients without chorea (84% vs 67%; ATE=0.167, *P*=0.071). Among those who stopped working, more than half of the patients with and without chorea (57% vs 55%; ATE=0.015, *P*=0.871) stopped due to difficulties completing everyday tasks. Compared to those without chorea, more patients exhibiting symptoms of chorea had stopped working due to cognitive symptoms worsening (53% vs 31%; ATE=0.218, *P*=0.011) or due to the onset of motor symptoms (52% vs 25%; ATE=0.278, *P*=0.002; **Figure 1**). HD patients with chorea also discontinued work significantly more frequently due to worsening of behavioral symptoms than did patients without chorea (37% vs 21%; ATE=0.160, *P*=0.045; **Figure 1**).

Physicians reported impact of HD on working hours for 143 employed patients (with chorea n=90, without chorea n=53). Although there was no significant difference between the two groups in terms of having to reduce work hours due to HD, over a third of all employed patients had experienced the need to reduce their hours (41% vs 33%; ATE=0.087, *P*=0.289).

**Figure 2.**
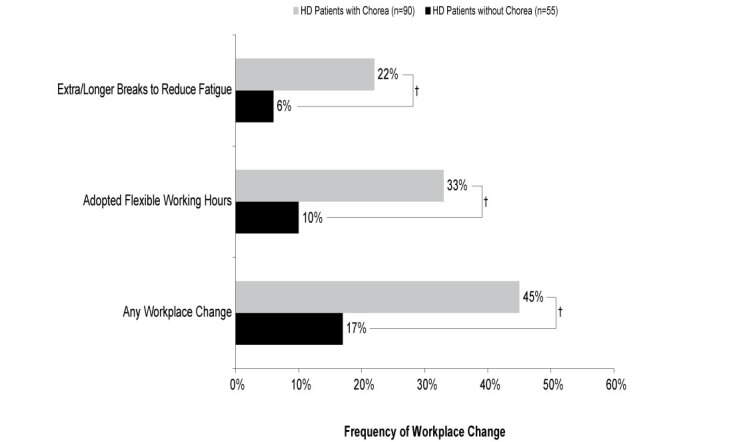
Changes in the Workplace Due to HD Abbreviations: HD, Huntington’s disease. †*P*<0.01.

The need for workplace changes were reported for 145 employed patients. Over two-fifths of patients with chorea (n=90) required changes in their workplace and required these changes more frequently than those without chorea (n=55) (45% vs 17%; ATE=0.279, *P*<0.001; **Figure 2**). Flexible working hours and extra/longer breaks to reduce fatigue were among the most common changes required to the workplace for HD patients with chorea; both were significantly more frequent in patients with chorea than in those without chorea (flexible working hours, 33% vs 10%; ATE=0.233, *P*<0.001; extra/ longer breaks to reduce fatigue, 22% vs 6%; ATE=0.156, *P*=0.003).

### Impact of Chorea on Self-care Activities (IPWRA Analysis)

**Figure 3.**
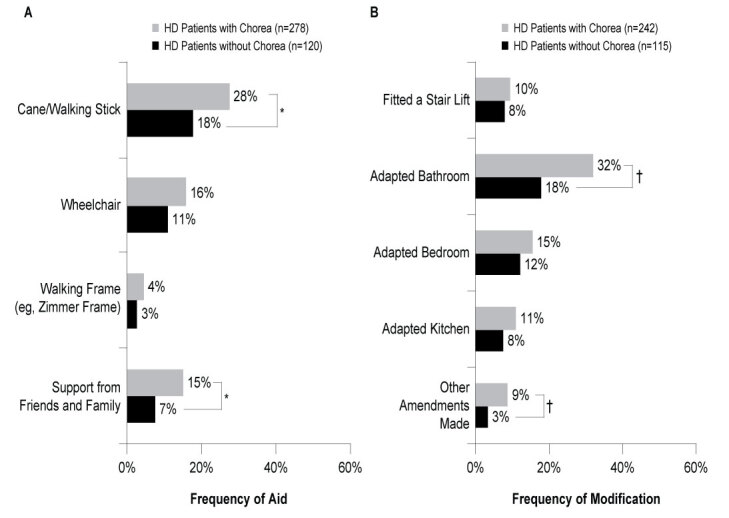
Need for Aid (A) and Need for Home Modification (B) Due to HD Abbreviations: HD, Huntington’s disease. *P<0.05; †*P*<0.01.

Physicians were aware of the requirement for mobility aids for 398 HD patients including 278 with chorea and 120 without chorea. Analysis found that HD patients with chorea required aid to help them get around significantly more frequently than those without chorea (55% vs 34%; ATE=0.205, *P*<0.001). Significantly more patients with chorea required a cane/walking stick than did those without chorea (28% vs 18%; ATE=0.100, *P*=0.032), and twice as many patients with chorea (versus those without) required support from friends and family (15% vs 7%; ATE=0.076, *P*=0.030; **Figure 3A**).

Physicians were aware of changes to home accommodations for 357 of the HD patients in the sample, 242 with chorea, 115 without chorea. HD patients with chorea also required modifications to their homes more frequently than those without chorea (39% vs 22%; ATE=0.177, *P*<0.001). Almost a third of patients with chorea had adapted a bathroom to accommodate their HD, significantly more than those without chorea (32% vs 18%; ATE=0.142, *P* =0.001; **Figure 3B**).

**Figure 4.**
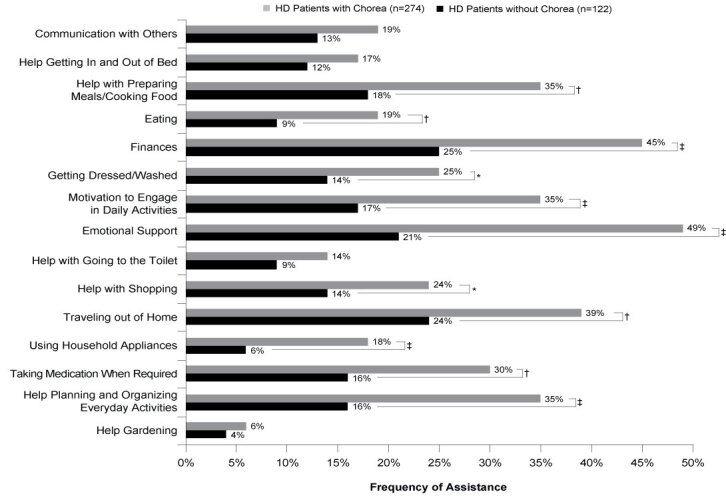
Self-care Activities Requiring Assistance Abbreviations: HD, Huntington’s disease. *P<0.05; †*P*<0.01; ‡*P*<0.001.

Physicians were aware of the assistance needs for everyday tasks of 396 patients in the sample, 274 with chorea, 122 without chorea. Almost all patients with and without chorea included in the analysis required some form of assistance with self-care activities (99% vs 96%; ATE=0.010, *P*=0.433). Significantly, over twice as many patients with chorea required emotional support compared with those without chorea (49% vs 21%; ATE=0.276, *P*<0.001), and almost twice as many patients with chorea required assistance with their finances (45% vs 25%; ATE=0.196, *P*<0.001). Across 11 of the 15 activities assessed, patients with chorea required assistance significantly more frequently than those without chorea (**Figure 4**).

### Impact of Chorea on Health-care Resource Use (IPWRA Analysis)

**Figure 5.**
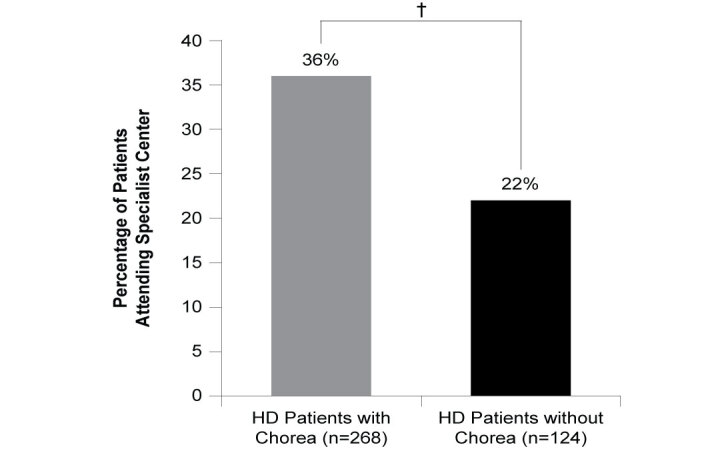
Attendance at Specialist Center Abbreviations: HD, Huntington’s disease. †*P*<0.01.

**Figure 6.**
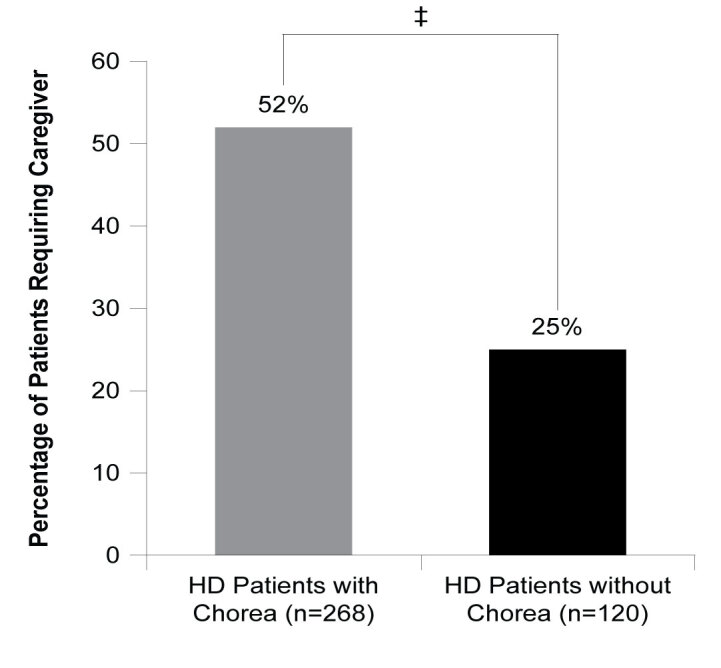
Requirement for Caregiver To Assist with Daily Living in Patients with and without Chorea Abbreviations: HD, Huntington’s disease. ‡*P*<0.001.

An analysis of patients known to be consulting for HD for at least 12 months (n=62) showed HD patients with chorea more frequently consulted with their neurologist for the management of their HD than those without chorea symptoms. In the 12 months prior to data collection, HD patients with chorea consulted with their neurologist a weighted mean of 3.4 times a year (n=53) versus 2.3 times a year (n=9) for those without chorea (ATE=1.113, *P*=0.001). HD patients with chorea (n=270) were more likely to visit a specialist center for the management of their HD compared to those without chorea (n=122) (35% vs 24%; ATE=0.112, *P*=0.022), excluding patients whose physician did not know whether the patient was attending a specialist center (n=35; **Figure 5**). Where caregiver requirement was known (n=386), HD patients with chorea were more likely to require a caregiver who was responsible for their daily needs, with over half of patients who were experiencing chorea requiring a caregiver compared with just over one-quarter of patients without chorea (51% vs 28%; ATE=0.229, *P*<0.001; **Figure 6**).

## DISCUSSION

The results of this real-world survey of physicians and their consulting patients with HD demonstrate that those with chorea experienced greater impairment in employment and self-care activities and greater HCRU than those without chorea. Previous studies have demonstrated that chorea interferes with daily functioning, increases the risk of injury, and has been reported by patients to negatively impact HRQoL, anxiety, and positive affect.^4,17,26^ While we recognize that the cognitive and neuropsychiatric aspects of HD substantially impact aspects such as self-care activities, employment, and HCRU, this study focuses on chorea, a prominent symptom of HD. Motor symptoms associated with HD were more frequently reported as a reason to stop working by patients with chorea as opposed to those without chorea, along with worsening cognitive and behavioral symptoms. Each reason was more frequently reported by patients with chorea than those without, suggesting a higher frequency of these symptoms in the workplace among patients with chorea. Although it was not significant, the proportion of HD patients who had stopped working (on long-term sick leave/retired/unemployed) as a result of their HD was higher in patients with chorea (84% vs 67%); among those who were still working, one-third required changes to their workplace to enable them to work comfortably. These workplace changes were required more frequently for patients with chorea than those without chorea, implying that chorea can make it difficult for individuals to continue working. This supports previous data indicating that chorea may have an impact on a patient’s ability to perform in the workplace.^5^ As patients with chorea required more/longer breaks and more flexible working hours than patients without chorea, treatment of HD chorea may be able to improve these patients’ productivity and ability at the workplace. In this study, HD patients with chorea reported needing mobility aids, such as a cane/walking stick, more frequently than those without chorea. Previous studies have shown that chorea can negatively impact a patient’s quality of life, affecting their daily functioning and potentially increasing their chance of injury.^5^ Home modifications such as an adapted bathroom or bedroom were more frequently reported for patients with chorea than those without chorea, implying that this symptom can profoundly affect everyday life for these patients.

Caregiver impact was assessed by examining the proportion of patients in each group (with chorea vs without chorea) who required a caregiver for assistance with daily activities. Those with chorea required a caregiver significantly more frequently than those without chorea, with almost twice as many patients with chorea requiring a caregiver. This shows that chorea associated with HD not only affects patients but also burdens caregivers as well. Similarly, patients with chorea had increased HCRU compared to those without. Within the 12 months prior to data collection, patients with chorea were found to consult their neurologist significantly more frequently than those without chorea, demonstrating higher HCRU in patients with chorea versus in those without.

Several limitations should be considered in the evaluation of our findings. First, patients who participated in the study may not reflect the general HD population, as the level of care could vary among patients depending on the providers or centers where they were treated. Therefore, our findings may not be fully generalizable to all HD patients. Furthermore, a number of factors, disease duration, and disease severity could impact the level of care. This study did not examine disease duration or other measures of disease severity, such as total functional capacity; cytosine, adenine, guanine repeats; impairments of voluntary movements; and cognitive deficits. Therefore, the association that HD patients with chorea need additional support for daily activities may not definitively be explained by the presence of chorea alone. Given the data available and outcomes analyzed, IPWRA methods were applied to adjust the cohorts based on potential confounding factors prior to comparisons, which should have limited some of their effect on the findings, thereby representing a pragmatic approach to the analysis. Second, recall bias might also have affected the responses of patients/caregivers and physicians to the questionnaires, which is a common limitation of surveys. Although it has been reported that ~90% of HD patients experience chorea, only approximately two-thirds of patients were reported to have chorea in this study. The reason for this discrepancy could be attributed, in part, to the fact that neurologists were asked to identify HD with or without chorea partly from retrospective chart review, allowing for possible error when reporting chorea. Data collected at the time of each patient’s appointment is expected to mitigate recall bias. Third, it was not possible to confirm that no information exchange occurred between physicians and their patients at the time of data collection; however, this is likely to be mitigated by methods in our research design that ensured physicians and staff were unaware of patient/caregiver responses. Finally, the definition of chorea may have been overly broad in this study; in particular, movements related to clumsiness may not have been related to chorea. Despite such limitations, real-world studies play an important part in highlighting areas of concern and disease burden that are not addressed in clinical trials.

## CONCLUSION

This real-world data collection from HD patients highlights the burden that chorea can have on everyday life. Chorea can negatively impact the ability to continue working, impair day-to-day activities, and increase HCRU. Findings from this analysis suggest that alleviating chorea has the potential to improve day-to-day functioning for these patients, enabling them to remain employed and reduce caregiver and health-care resource burdens.

### Author Roles

DOC and RGI contributed to study concept and design, and interpretation of results. JD, JM, and CJ contributed to study concept and design, data collection, data analysis, interpretation of results, and drafting of the manuscript. All authors contributed to the review, critical revision, and final approval of the manuscript.

### Disclosures

DOC received grant support from the National Institutes of Health’s National Institute of Neurological Disorders and Stroke, Michael J. Fox Foundation, Huntington Disease Society of America, Vaccinex, AbbVie, and Auspex Pharmaceuticals; and received consulting fees from Teva Neuroscience, Lundbeck, Acadia, and AbbVie. JD, JM and CJ report no disclosures. RGI is an employee of Teva Pharmaceuticals. All authors confirm that we have read the Journal’s position on issues involved in ethical publication and affirm that this work is consistent with those guidelines.

## Supplementary Material

Supplementary Material
