# Supplementary material for: Impact of Chorea on Self-care Activity, Employment, and Health-care Resource Use in Patients with Huntington’s Disease
Source: J Health Econ Outcomes Res. 2021 Jun 21;8(1):99–105. doi: 10.36469/001c.24620 (PMC8216765; doi:10.36469/001c.24620)
Supplement: Supplementary Material [file jheor_2021_8_1_24620_62897.pdf]

Journal of Health Economics and Outcomes Research

Supplementary Online Material

Claassen DO, DeCourcy J, Mellor J, Johnston C, Iyer RG. Impact of chorea on self-care activity, employment, and health-care resource use in patients with Huntington's disease. *JHEOR*. 2021;8(1):99-105. doi:10.36469/jheor.2021.24620

Supplemental Table 1. Descriptive Patient Demographics
Supplemental Figure 1. Sample Flow Diagram

This supplementary material has been provided by the authors to give readers additional information about their work.

This is an open-access article distributed under the terms of the Creative Commons Attribution 4.0 International License (CCBY-4.0). View this license's legal deed at http://creativecommons.org/licenses/by/4.0 and legal code at http://creativecommons.org/licenses/by/4.0/legalcode for more information.

Table S1. Descriptive Patient Demographics

	All HD Patients (n=427)	HD Patients with Chorea (n=287)	HD Patients without Chorea (n=140)	*P*-value (HD patients with chorea vs HD patients without chorea)
**Age, Years**				
Mean (SD)	49.8 (13.1)	50.9 (11.9)	47.5 (15.1)	0.013 (TT)
**Sex, n (%)**				
Male	272 (64)	182 (63)	90 (64)	0.915 (FE)
Female	155 (36)	105 (37)	50 (36)	
**BMI, kg/m^2**				
Mean (SD)	24.9 (3.9)	24.8 (3.7)	25.0 (4.3)	0.745 (TT)
**Employment Status, n (%)**				
Working Full Time	84 (20)	48 (17)	36 (26)	0.038 (FE)
Working Part Time	70 (16)	47 (16)	23 (16)	1.000 (FE)
On Long-term Sick Leave	57 (13)	50 (17)	7 (5)	<0.001 (FE)
Homemaker	29 (7)	23 (8)	6 (4)	0.218 (FE)
Student	10 (2)	2 (1)	8 (6)	0.003 (FE)
Retired	60 (14)	42 (15)	18 (13)	0.659 (FE)
Unemployed	86 (20)	58 (20)	28 (20)	1.000 (FE)
Don't Know	31 (7)	17 (6)	14 (10)	

Abbreviations: BMI, body mass index; FE, Fishers exact test, HD, Huntington's disease; SD, standard deviation; TT, Student's t-test.

Figure S1. Sample Flow Diagram

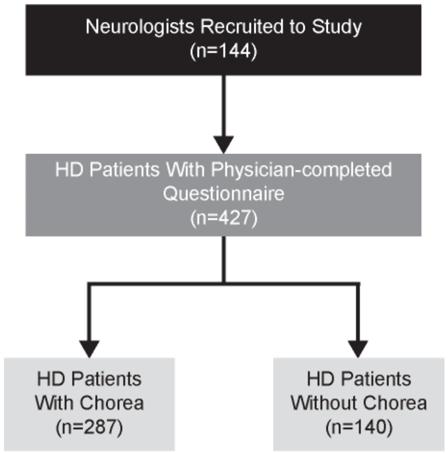

Abbreviations: HD, Huntington's disease.
